# Effects of green tea extract on overweight and obese women with high levels of low density-lipoprotein-cholesterol (LDL-C): a randomised, double-blind, and cross-over placebo-controlled clinical trial

**DOI:** 10.1186/s12906-018-2355-x

**Published:** 2018-11-06

**Authors:** Lin-Huang Huang, Chia-Yu Liu, Li-Yu Wang, Chien-Jung Huang, Chung-Hua Hsu

**Affiliations:** 1 Institute of Traditional Medicine, National Yang-Ming University, No. 155, Section 2, Linong St, Beitou District, Taipei City, Taiwan 112; 2 Branch of Linsen, Chinese Medicine, and Kunming, Taipei City Hospital, No. 530, Linsen North Road, Zhongshan District, Taipei City, 104 Taiwan; 3 Department of Endocrinology, Branch of Linsen, Chinese Medicine, and Kunming, Taipei City Hospital, No. 530, Linsen North Road, Zhongshan District, Taipei City, 104 Taiwan

**Keywords:** Obesity, Green tea extract, Epigallocatechin gallant, EGCG, Hyperlipidemia, LDL-C, Low density lipoprotein, Leptin

## Abstract

**Background:**

This study aims to examine the effects of green tea extract (GTE) supplement on overweight and obese women with high levels of low density lipoprotein-cholesterol (LDL-C).

**Methods:**

The randomized, double-blind, crossover and placebo-controlled clinical trial was conducted from August 2012 to December 2013. Seventy-three out of 90 subjects aged between 18 and 65 years, with body mass index (BMI) ≥ 27 kg/m^2^ and LDL-C ≥ 130 mg/dl were included in the analysis. The subjects were randomly divided into Groups A and B. Group A received GTE supplement treatment for the first 6 weeks, while Group B received placebo daily. After 6 weeks of treatment and 14 days of washout period, Group A switched to placebo and Group B switched to GTE treatment for 6 weeks. The reduction of LDL-C level between treatments was assessed as the outcome. Additionally, anthropometric measurements, plasma lipoproteins and hormone peptides of both groups were measure at the beginning of weeks 6, 8, and 14 after treatment.

**Results:**

Subjects treated with GTE (*n* = 73) for 6 weeks showed significant differences, with 4.8% (*p* = 0.048) reduction in LDL-C and 25.7% (*p* = 0.046) increase in leptin. However, there was no statistical difference in the levels of total cholesterol, triglyceride and high density lipoprotein between the GTE and placebo groups after treatments.

**Conclusions:**

This study shows that green tea extract effectively increases leptin and reduces LDL in overweight and obese women after 6 weeks of treatment even though there were no significant changes in other biochemical markers related to overweight.

**Trial registration:**

This clinical trial is registered with ClinicalTrials.gov: NCT02116517 on 17 April 2014. Retrospectively registered. The first patient enrolled in October 2012 and the study was completed December 2013.

**Electronic supplementary material:**

The online version of this article (10.1186/s12906-018-2355-x) contains supplementary material, which is available to authorized users.

## Background

Obesity is one of the leading preventable causes of death in the world. Due to its high prevalence rate worldwide, it was classified as a chronic disease in 1995 by the World Health Organization (WHO) [1, 2]. Obesity and high levels of low density lipoprotein-cholesterol (LDL-C) are the main causes leading to cardiovascular disease (CVD) [3], whereas high level of LDL-C is considered as an important risk factor for CVD in obese individual. It is known that CVD morbidity is one of the major causes of global deaths [4]. Hyperlipidemia is also one of the key risk factors for CVD [5]. Numerous studies have found that obesity puts a person at an increased risk for diseases such as hyperlipidemia and CVD [6]. However, some drugs used to reduce LDL-C levels and treat hyperlipidemia [7–11] have been reported to have negative side effects on liver function [11–13]. It is therefore, imperative to explore alternatives such as herbal medicine to lower LDL-C that possesses fewer side effects.

Green tea (*Camellia sinensis*; GT), a very popular drink in the world [14], has been studied extensively for its beneficial effects on preventing cardiovascular diseases [12] and metabolic diseases [15–18]. Green tea extract (GTE) was reported to counter insulin resistance and favorably alter fat metabolism [19]. Other benefits of green tea include being antioxidant, anti-hypertensive, and anti-inflammatory. In particular, it has been shown to increase energy expenditure [20] and enhance metabolic rate and fat-burning ability [21], so as to relieve the hyperlipidemia and hyperglycemia, and had been used for slimming or obesity control for a long time [22–27]. Polyphenols of green tea are considered as the main constitutions attributed to anti-lipid effects of green tea. The four major flavonoids, epicatechin (EC), epigallocatechin (EGC), epicatechingallate (ECG), and epigallocatechin gallate (EGCG), are the catechins among all the bioactive polyphenols. The efficacy of green tea may be attributed to the presence of catechin polyphenols, and it has been suggested that EGCG could be responsible for the various health effects associated with green tea [28].

Metabolic syndrome is an escalating global epidemic. Obesity is a metabolic disorder characterized by excess fat accumulation in the body. Due to unsafe medicines and inevitable side effects, reducing metabolic disorders is burdened with serious problems. Medicinal plants and derived products are becoming increasingly popular as natural alternatives to synthetic drugs for the treatment of hypercholesterolemia and hypertriglyceridemia in modern society. Obesity is highly associated with the development of hyperlipidemia and non-alcoholic fatty liver disease. Many natural or plant-derived compounds, including GTE and EGCG have been researched to understand their effects on weight control [19, 29], or hyperlipidemia [30, 31]. However, little is known about the extent to which GTE can affect obese women with high LDL-C levels. Recently, Rocha et al. reported that GTE ameliorates the metabolic dysfunction of adipose tissue, that is induced by obesity, through the AMP-activated protein kinase (AMPK)-regulated pathway [32].

Men and women with the same degree of overweight show differences in that men have higher triglyceride, fasting glucose, insulin levels as well as blood pressure. There is also a difference in the distribution of adipose tissue, with women having more body fat [33]. Hence gender was stratified in this study.

We hypothesized that GTE would help in lowering LDL-C levels in overweight and obese women, and, therefore, investigated the anti-obesity effect of GTE on these women by focusing on both the biochemical and physiological responses in a randomized, cross-over clinical trial.

## Methods

### Study design and participants

This trial was conducted from August 2012 to December 2013 in Taipei City Hospital, Taiwan. Among 236 registered obese women screened at our outpatient clinic, a total of 90 participants were enrolled. Inclusion criteria for participants included the following: ethnically Taiwanese, female, age between 18 and 65 years, BMI ≥ 27 kg/m^2^, and LDL-C ≥ 130 mg/dl. Exclusion criteria included any of the following: (1) aminotransferases aspartate or aminotransferases alanine > 80 IU/L and serum creatinine > 1.8 mg/dl, (2) lactating or pregnant women, (3) a prior history of heart failure, acute myocardial infarct, stroke or heavy injuries, and (4) any other conditions not suitable for trial as evaluated by the physician-in-charge. Authors of this study had access to information that could identify individuals after data collection and all patients gave written consent by signing a consent form.

**Fig. 1:**
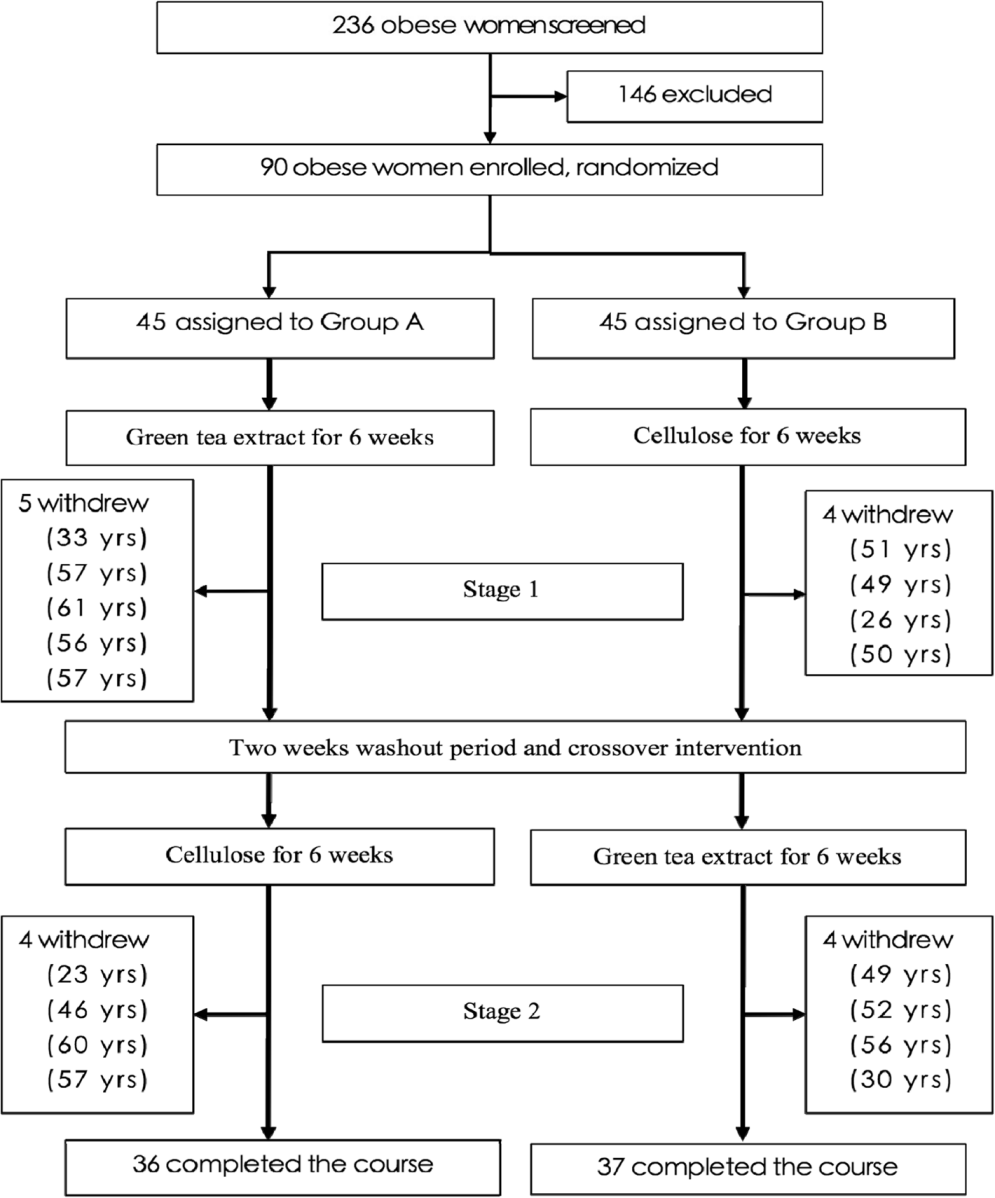
Trial Profile and Design

The participants were randomly divided into Group A and B. In the first stage, Group A received GTE supplement treatment while Group B received placebo daily. The second stage started after 14 days of washout, where Group A was switched to placebo and Group B to GTE for 6 weeks (Fig. 1). The protocol was approved by the Human Ethics Committee of the hospital. The patients were instructed to maintain an isocaloric diet and in accordance to their previous eating habit during the study period. Every subject had to come to the hospital once a week for blood sampling and for us to assess if they adhered to the prescribed GTE or placebo. All subjects were free to withdraw at any time during the course of the study.

### Randomization and blindness

All participants were randomly assigned to either placebo or GTE treatment. A random number between 0.0 and 0.99 was generated by the computer program for each subject. Those given random numbers between 0.0 and 0.49 were assigned to the treatment group to receive GTE, while others with numbers between 0.50 and 0.99 were assigned to the placebo group to receive cellulose, during stage 1. To ensure that the capsules looked and smelled identical, opaque capsules were used to contain either GTE or placebo (cellulose). They were administered to the participants by a research assistant who was also blinded to the contents of the capsules. Characteristics of all the participants at baseline are shown in Table 1.

**Table 1:** Characteristics of Participants at Baseline

Characteristics	Group A *n* = 36 mean (SD)	Group B *n* = 37 mean (SD)	*p* value
Anthropometric data			
Age, years	53.1 (11.2)	56.8 (7.9)	0.10
Weight, kg	70.6 (11.4)	67.8 (9.1)	0.25
Height, cm	156.0 (4.6)	155.7 (5.1)	0.79
Body Mass Index, kg/m^2^	29.1 (4.9)	27.9 (3.3)	0.26
Waist Circumference, cm	89.7 (12.4)	86.6 (9.5)	0.23
Hip Circumference, cm	100.8 (8.8)	99.5 (6.6)	0.48
Waist hip ratio	0.9 (0.1)	0.9 (0.1)	0.24
Biochemical data			
Fasting blood sugar, mg/dL	105.2 (23.8)	114.9 (49.0)	0.29
Total cholesterol, mg/dL	218.8 (42.4)	229.6 (30.3)	0.21
Triglyceride, mg/dL	147.4 (57.4)	152.6 (57.7)	0.70
High density lipoprotein (HDL), mg/dL	50.9 (12.1)	53.8 (15.3)	0.38
Low density lipoprotein (LDL), mg/dL	142.3 (37.2)	149.3 (23.6)	0.34
Hormone peptides			
Insulin, IU/L	17.3 (14.4)	16.0 (10.9)	0.66
HOMA-IR index	4.7 (5.3)	4.7 (3.8)	0.94
Adiponectin, ug/mL	12.1 (7.7)	14.7 (8.7)	0.18
Ghrelin, pg/mL	555.9 (330.4)	523.0 (298.5)	0.66
Leptin, ng/mL	18.5 (13.9)	17.3 (10.6)	0.67
Apolipoprotein A1, mg/dL	165.8 (29.2)	178.2 (38.1)	0.12
Apolipoprotein B100, mg/dL	117.8 (25.6)	120.8 (15.2)	0.54

### Preparation of samples and treatment

The GTE samples, extracted from dried green tea leaves, were procured from the Tea Research and Extension Station, Taiwan. It was manufactured by a standard procedure and came with a certificate of analysis. The control group was given placebo which was pure microcrystalline cellulose. The participants were asked to take one capsule 30 min after meal, three times a day for 6 weeks in each stage. Daily intake of GTE compounds is listed in Table 2, among which EGCG amounted to 856.8 mg.

**Table 2:** The composition of green tea extracts

Component	% in weight	Daily dose (in mg)
EGCG (Epigallocatechin gallate)	57.12	856.8
ECG (Epicatechingallate)	15.74	236.1
EGC (Epigallocatechin)	7.70	115.5
EC (Epicatechin)	4.80	71.9
GCG (Gallocatechingallate)	4.25	63.7
GC (Gallocatechin)	< 0.07	< 1.05
Caffeine	< 0.07	< 1.05
Cellulose	10.3	155.0

HOMA-IR = insulin [mIU/L] × glucose [mmol/L]/22.5, and values exceeding 2.25 would be considered as insulin resistance

HOMA-IR index = insulin (μU/mL) × glucose (mmol/L)/22.5

Glucose 1 mg/dL = 0.0555 mmol/L (http://www.endmemo.com/medical/unitconvert/Glucose.php)

### Outcome measurements

As Table 1 shows, reduction of LDL-C level between two different treatments was used as one of the major outcome measurements. Additionally, plasma lipoproteins such as triglyceride, cholesterol, HDL-C and LDL, as well as hormone peptides of both groups were measured at the baseline, the 6th, 8th and 14th week after treatments. All measurements were made 8–9 h after an overnight fast using standardized methods and were repeated throughout the trial. All the participants had their measurements done with their undergarments and a hospital gown. A wall-mounted stadiometer was used to measure their height to the nearest 0.1 cm, their weight was measured on a calibrated balance beam scale to the nearest 0.1 kg, and BMI was calculated according to the standardized formula: BMI = BW / height^2^ (kg/m^2^). Waist circumflex (WC) was measured by the mid-way between the lateral lower rib margin and the iliac crest. The demographic data was collected simultaneously.

### Obesity-related hormone peptides

The obesity-related hormone peptides, including leptin, insulin, ghrelin, adiponectin, apolipoprotein (Apo) A1 and apolipoprotein B100 [7–35] were measured by collecting blood samples in the morning after fasting for 8–9 h. After blood was drawn and centrifuged at 4 °C, a milliliter of aliquot serum was added to the samples and then quick-frozen at − 80 °C for subsequent radioimmunoassay concentration analysis. The leptin was measured by Millipore Human Leptin assay kit (Millipore, St. Charles, MO, USA), using the I^125^-labled human leptin antiserum with a sensitivity of 0.5 ng/ml for a 100-μL sample. The ghrelin and adiponectin were identified by the same process as for leptin, with the only difference that I^125^-labled antibodies were specific to ghrelin or adiponectin, using Ghrelin, and Adiponectin RIA kits (Millipore, St. Charles) with the sensitivity of 93 pg/ml and 1 ng/ml, respectively. The levels of insulin in serum were determined using BioSource INS-IRMA Kits (BioSource Europe S.A., Nivelles, Belgium). The respective coefficients of variation were 3.1% for intra-assay and 4.9% for inter-assay. The parameter of sensitivity was 0.5 ng/ml. It was reported that a differentiation exceeding 10% coefficients of variation was found between replicated results of the sample. The homeostasis model assessment of insulin resistance index (HOMA-IR), used to evaluate level of insulin resistance, was calculated with the following formula: HOMA-IR = insulin [mIU/L] × glucose [mmol/L]/22.5. Insulin resistance was indicated when HOMA-IR values exceeded 2.25. The Apo AI and Apo B-100 levels of participants were detected by Immunoturbidimetric assay (K-assay, Kamiya Biomedical Company, Seattle, USA).

### EGCG dose analysis

The sample was extracted by sonication with 100 ml of 50% methanol for 10 min, and 2 ml of the extract was centrifuged at 10,000 rpm (Eppendorf Centrifuge 5402, MI, USA) for another 10 min. After filtrating the supernatant with a 0.22-μm syringe filter (Millipore, Bedford, MA, USA) 20 μl of the filtrate was injected into the High Performance Liquid Chromatography (HPLC) system. HPLC analysis used in this study was performed by a Hitachi 7000 series module equipped with a photodiode array detector and the wavelength was set at 273 nm. Catechin, epicatechin and EGCG were separated individually from the extract using a Merck Purospher STAR C-18 (50*4.6 mm. i.d., 5 μm). The flow rate of the mobile phase was 0.8 ml/min. All samples were analyzed at room temperature (25 ± 1 °C).

### Statistical analysis

All the data was analyzed using SPSS software (version 16, Chicago, IL). Student *t*-test was employed to examine the main outcomes, demographic data, and other measurements. Paired *t*-tests were utilized to examine differences of subjects within groups at 0 to 6 weeks in stage 1 and weeks 8 to 14 in stage 2. All *p* values were two-tailed and the α level of significance was set at 0.05.

## Results

Among all the data shown in Table 3, most of the anthropometric data such as weight, body mass index, WC, and waist hip ratio were not significantly different between treatments in stages 1 and 2, by both paired or non-paired *t*-test. Only the HC of Group B significantly decreased from 100.5 cm on the 56th day to 98.9 cm on the 98th day in stage 2, with the reduction rate at 1.6%.

**Table 3:** Measurements at different stages and the days after intervention

Measurements	Stage	Days	Group A *n* = 36 mean (SD)	Group B *n* = 37 mean (SD)	*p*-value
Weight, kg	1	0	70.6 (11.4)	67.8 (9.1)	0.25
	1	42	70.4 (11.5)	67.7 (9.2)	0.28
*p*-value for paired *t*-test			0.45	0.58	
	2	56	70.5 (11.2)	68.0 (9.3)	0.30
	2	98	68.4 (15.5)	67.6 (9.3)	0.80
*p*-value for paired *t*-test			0.27	0.13	
Body Mass Index, kg/m^2^	1	0	29.1 (4.9)	27.9 (3.3)	0.26
	1	42	28.9 (4.9)	27.9 (3.3)	0.29
*p*-value for paired *t*-test			0.44	0.60	
	2	56	29.0 (4.8)	28.0 (3.4)	0.32
	2	98	28.1 (6.6)	27.8 (3.4)	0.81
*p*-value for paired *t*-test			0.27	0.11	
Waist Circumference (WC), cm	1	0	89.7 (12.4)	86.6 (9.5)	0.23
	1	42	90.0 (11.3)	87.2 (8.2)	0.22
*p*-value for paired *t*-test			0.66	0.40	
	2	56	89.2 (10.7)	87.8 (9.6)	0.55
	2	98	89.8 (10.1)	87.4 (9.5)	0.29
*p*-value for paired *t*-test			0.27	0.57	
Hip Circumference (HC), cm	1	0	100.8 (8.8)	99.5 (6.6)	0.48
	1	42	101.1 (8.7)	99.5 (6.6)	0.38
*p*-value for paired *t*-test			0.57	0.93	
	2	56	100.7 (8.7)	100.5 (7.2)	0.91
	2	98	101.0 (9.0)	98.9 (7.6)	0.29
*p*-value for paired *t*-test			0.38	**0.01**	
Waist hip ratio	1	0	0.89 (0.07)	0.87 (0.06)	0.24
	1	42	0.89 (0.06)	0.88 (0.06)	0.36
*p*-value for paired *t*-test			0.78	0.28	
	2	56	0.88 (0.05)	0.87 (0.06)	0.41
	2	98	0.89 (0.05)	0.88 (0.06)	0.65
*p*-value for paired *t*-test			0.44	0.21	
Fasting blood sugar, mg/dL	1	0	105.2 (23.8)	114.9 (49.0)	0.29
	1	42	102.8 (18.7)	106.4 (28.6)	0.53
*p*-value for paired *t*-test			0.37	**0.047**	
	2	56	103.4 (22.0)	103.1 (22.0)	0.96
	2	98	103.1 (26.2)	106.2 (26.9)	0.62
*p*-value for paired *t*-test			0.90	0.23	
Total cholesterol, mg/dL	1	0	218.8 (42.4)	229.6 (30.3)	0.21
	1	42	213.3 (41.4)	226.2 (25.9)	0.12
*p*-value for paired *t*-test			0.13	0.47	
	2	56	220.3 (46.3)	224.8 (31.9)	0.62
	2	98	220.3 (40.3)	217.6 (27.7)	0.74
*p*-value for paired *t*-test			1.00	0.05	
Triglyceride, mg/dL	1	0	147.4 (57.4)	152.6 (57.7)	0.70
	1	42	144.4 (59.7)	152.5 (66.1)	0.58
*p*-value for paired *t*-test			0.72	1.00	
	2	56	134.9 (53.4)	138.5 (49.5)	0.77
	2	98	142.8 (47.3)	150.4 (53.3)	0.52
*p*-value for paired *t*-test			0.32	0.10	
High density lipoprotein (HDL), mg/dL	1	0	50.9 (12.1)	53.8 (15.3)	0.38
	1	42	50.1 (10.5)	53.6 (12.1)	0.19
*p*-value for paired *t*-test			0.39	0.88	
	2	56	50.0 (11.2)	53.6 (12.5)	0.19
	2	98	50.4 (11.2)	53.7 (12.5)	0.25
*p*-value for paired *t*-test			0.64	0.98	
Low density lipoprotein (LDL), mg/dL	1	0	142.3 (37.2)	149.3 (23.6)	0.34
	1	42	135.3 (39.8)	145.8 (22.8)	0.17
*p*-value for paired *t*-test			0.05	0.38	
	2	56	142.7 (41.6)	145.5 (29.4)	0.74
	2	98	141.9 (34.4)	136.9 (26.2)	0.49
*p*-value for paired *t*-test			0.84	**0.01**	
Insulin, IU/L	1	0	17.3 (14.4)	16.0 (10.9)	0.66
	1	42	13.9 (6.6)	15.9 (9.3)	0.31
*p*-value for paired *t*-test			0.14	0.97	
	2	56	15.3 (6.2)	15.2 (9.4)	0.94
	2	98	16.3 (7.7)	16.5 (14.3)	0.95
*p*-value for paired *t*-test			0.30	0.38	
HOMA-IR index	1	0	4.7 (5.3)	4.7 (3.8)	0.94
	1	42	3.4 (1.6)	4.3 (3.4)	0.19
*p*-value for paired *t*-test			0.17	0.63	
	2	56	3.8 (1.7)	3.9 (2.4)	0.89
	2	98	4.2 (2.3)	4.5 (4.4)	0.72
*p*-value for paired *t*-test			0.26	0.25	
Adiponectin, ug/mL	1	0	12.1 (7.7)	14.7 (8.7)	0.18
	1	42	13.3 (6.0)	16.1 (8.3)	0.10
*p*-value for paired *t*-test			0.14	**0.02**	
	2	56	13.3 (8.6)	15.8 (7.9)	0.20
	2	98	10.8 (4.8)	14.6 (9.0)	**0.03**
*p*-value for paired *t*-test			**0.02**	0.15	
Ghrelin, pg/mL	1	0	555.9 (330.4)	523.0 (298.5)	0.66
	1	42	481.9 (173.0)	531.5 (225.8)	0.30
*p*-value for paired *t*-test			0.20	0.87	
	2	56	427.9 (146.6)	450.1 (149.3)	0.52
	2	98	357.5 (90.4)	370.5 (119.8)	0.60
*p*-value for paired *t*-test			**0.01**	**0.01**	
Leptin, ng/mL	1	0	18.5 (13.9)	17.3 (10.6)	0.68
	1	42	19.3 (14.1)	15.1 (7.2)	0.12
*p*-value for paired *t*-test			0.75	0.15	
	2	56	15.1 (9.4)	14.1 (7.1)	0.60
	2	98	14.4 (10.2)	14.5 (7.7)	0.97
*p*-value for paired *t*-test			0.57	0.72	
Apolipoprotein A1, mg/dL	1	0	165.8 (29.2)	178.2 (38.1)	0.12
	1	42	166.0 (33.7)	171.6 (34.4)	0.48
*p*-value for paired *t*-test			0.97	0.16	
	2	56	175.6 (34.5)	184.8 (35.6)	0.27
	2	98	174.1 (36.8)	180.5 (34.2)	0.44
*p*-value for paired *t*-test			0.69	0.36	
Apolipoprotein B100, mg/dL	1	0	117.8 (25.6)	120.8 (15.2)	0.54
	1	42	117.5 (28.2)	120.4 (18.3)	0.61
*p*-value for paired *t*-test			0.91	0.88	
	2	56	123.8 (31.4)	125.1 (21.8)	0.85
	2	98	121.9 (27.7)	118.9 (19.1)	0.58
*p*-value for paired *t*-test			0.55	**0.02**	

In stage 1, fasting blood sugar and plasma lipoproteins of Group A did not change significantly. In contrast, fasting blood sugar and adiponectin of Group B significantly decreased on the 42th day of treatment, only by the paired *t*-test. The fasting blood sugar means (SD) of Group B reduced from 114.9 mg/dL (49.0) to 106.4 mg/dL (28.6) on the 42th day of treatment. There was a significant 7.4% reduction in fasting blood sugar (*p* = 0.047, paired t-test) in Group B as compared with that of Group A (2.3%). Except adiponectin, the hormone peptides of Group A and Group B were not significantly different after treatments in this stage. The means (SD) of adiponectin increased from 14.7 μg/mL (8.7) to 16.1 μg/mL (8.3) on the 42th day of treatment, showing a 9.5% increase which was higher than that of Group A (9.9%).

In stage 2, Group A was given placebo and Group B received GTE. Reductions were observed in several biomarkers, i.e. LDL, adiponectin, ghrelin and apolipoprotein B100. In Group B, LDL at this stage showed a 5.9% reduction, means (SD) decreasing from 145.5 mg/dL (29.4) on the 56th day to 136.9 mg/dL (26.2) on the 98th day (*p* = 0.01). The adiponectin of Group A showed an 18.8% reduction from 13.3 μg/mL (8.6 for SD) on the 56th day to 10.8 μg/mL (4.8 for SD) on the 98th day, as compared to a 7.6% reduction in Group B (*p* = 0.02). In Groups A and Group B, ghrelin level significantly decreased, showing a reduction of 19.7% (*p* = 0.01, paired *t*-test) and 17.7% (*p* = 0.01), respectively. There was also a 5.0% reduction in Apolipoprotein B100 for Group B, from 125.1 mg/dL (21.8 for SD) to 118.9 mg/dL (19.1 for SD) (*p* = 0.02).

The double-blind, placebo-controlled, cross-over design study showed that there were no statistical difference in the levels of BMI, fasting blood sugar, total cholesterol, triglyceride, high density lipoprotein, adiponectin and ghrelin etc. between the GTE and placebo groups after treatments. However, it especially showed a significant 25.7% increase in leptin and 4.8% decrease in LDL-C levels over the trial period, with the *p* values being 0.046 and 0.048 respectively (Table 4) compared to the control group.

**Table 4:** Outcome of epigallocatechin gallate and cellulose after 6 weeks of treatment

*n* = 73	Green tea extract	cellulose	
Cases reductions, %	mean (SD)	mean (SD)	*p* value
Weight, kg	−0.5 (2.5)	−1.5 (10.7)	0.41
Body Mass Index, kg/m^2^	−0.5 (2.5)	−1.5 (10.7)	0.41
Waist Circumference (WC), cm	0.2 (5.0)	0.9 (4.5)	0.36
Hip Circumference (HC), cm	−0.6 (3.2)	0.2 (2.6)	0.11
Waist hip ratio	0.8 (5.0)	0.7 (4.1)	0.91
Fasting blood sugar, mg/dL	1.2 (11.9)	−2.0 (12.9)	0.12
Total cholesterol, mg/dL	−2.3 (9.7)	0.3 (12.3)	0.15
Triglyceride, mg/dL	7.5 (32.6)	9.8 (41.0)	0.71
High density lipoprotein, mg/dL	−0.1 (10.2)	1.8 (14.6)	0.35
Low density lipoprotein, mg/dL	−4.8 (14.6)	0.5 (17.3)	**0.048**
Insulin, IU/L	4.5 (47.4)	11.8 (47.0)	0.35
HOMA-IR index	7.1 (54.4)	9.3 (42.7)	0.78
Adiponectin, ug/mL	15.2 (68.8)	6.9 (42.2)	0.38
Ghrelin, pg/mL	−6.0 (39.0)	1.4 (39.7)	0.26
Leptin, ng/mL	25.7 (103.8)	−1.02 (44.9)	**0.046**
Apolipoprotein A1, mg/dL	0.4 (20.5)	−1.3 (14.4)	0.55
Apolipoprotein B100, mg/dL	−2.0 (12.6)	0.2 (14.8)	0.35

(+ means increase and − means reduction)

Green tea consumption in the form of EGCG, GTE or catechins by subjects with high BMI may decrease body weight, BMI, waist and hip circumference and improve blood lipid profile, glucose homeostasis as well as decrease inflammatory markers. However, the results are not consistent by showing either an improvement or no effect.

## Discussion

There have been many studies that reported on the beneficial effects of GTE on a person’s health including weight loss. Our study showed significant increase in leptin and decrease in LDL-C levels in patients who received GTE treatment.

With an increase in obesity prevalence, many alternative treatments for weight control are developed [34]. Studies argued that GTE appeared to have anti-obesity and anti-diabetic effects. However, in this double-blind, placebo-controlled, cross-over design study, the results revealed that green tea extract with a high dose of EGCG was not able to produce a significant decrease in body weight, BMI or fasting blood sugar levels in obese Taiwanese women within the trial period although in many human studies there have been reported decrease in body weight and body fat [35].

GTE has shown to increase fat oxidation and energy expenditure due to its high concentration of EGCG [36]. EGCG inhibits catechol-o-methyltransferase (COMT) which is an enzyme that causes the degradation of norepinephrine. This in turns results in increased lipolysis and fat oxidation [37]. It has been suggested that the COMT single nucleotide polymorphism (SNP rs4680) is very common in Asians and alters sensitivity to EGCG, which leads to a greater energy expenditure and body fat loss due to more effective supplementation. Therefore it is important to consider the ethnicity when conducting this kind of study as better results are obtained amongst the Asian population in comparison to Caucasians [38].

Furthermore, leptin has a role in regulating lipid metabolism as it stimulates fatty acid oxidation and decreases triglyceride stores within fat cells in the liver as well as inhibiting lipogenesis and stimulates lipolysis in fat cells [40].

Once leptin is released by the adipose tissue, it is secreted into the bloodstream and circulated in proportion to body fat mass [39]. It provides the brain with information about the status of the body’s energy stores. There are several factors that influence the regulation of circulating leptin levels including food intake and glucose uptake which increase the levels of circulating leptin while exercise and increased age decrease the levels of circulating leptin [41].

Leptin crosses the blood-brain barrier and activates the leptin receptor, which causes an inhibitory effect on associated insulin stimulated metabolic pathways. Therefore treating adipocytes with leptin reduces insulin stimulation of carbohydrate and lipid metabolism as well as insulin stimulation of protein synthesis. Previous studies have found that leptin suppresses the LDL receptors (LDL-R) through the repressed expression of the SREBP2, which is a transcription factor of LDLR gene [39].

The LDLR gene is responsible for the production of LDL-R, which binds LDL particles. LDL primarily carry cholesterol in the blood. LDL-R binds to LDL circulating in the bloodstream and transports them into the cell. The LDL are broken down and the cholesterol is released. LDL-R are critical in regulating the amount of cholesterol in the blood [42].

Our study showed that there was a significant decrease in LDL levels after GTE consumption. It is consistent with a previous study that showed green tea decreasing absorption of lipids and proteins in the intestine and also activating the pathway to decrease gluconeogenesis and fatty acid synthesis [43]. It is, however, suggested that disruption of leptin receptors have no significant impact on target organs because its main effect is on the brain that then has the effect on glucose and lipid metabolism [44] and thus this study suggests that GTE is the main cause of the lowering of LDL levels.

There has been similar studies done in the past to determine whether green tea extracts have been advantageous in improving various biochemical markers related to obesity but this is one of the few studies that is designed with a crossover. This allows for accurate comparisons of biochemical markers within the same individuals during stages 1 and 2. Each individual serves as his or her own control, therefore decreasing the variability between the individuals. It is therefore not necessary to make any adjustments to variables such as dietary intake, levels of physical activity or post-menopausal changes. At the commencement of the trial, individuals were also instructed to follow their previous dietary habits throughout the course of the study as stated in the Method section. Another advantage of this crossover study is that it requires a smaller sample size compared to previous studies such as Samavat et al. [45] and Tian C et al. [46] having 936 and 19,471 participants respectively. With a smaller sample size, there runs a risk of participants withdrawing leaving little data to be analyzed.

This study presents with several limitations. Firstly, the short duration of the study might not have allowed sufficient time for significant changes to take place. When compared with previous studies such as Samavat et al. [45] and Tian C et al. [46] running over the duration of years. In retrospect, a study by Li [47] suggests that noticeable effects of tea only occur after 8 or more weeks of intervention. Even though participants were only exposed to GTE for 6 weeks, it caused a noticeable change in leptin and LDL levels. Secondly, the gender and ethnical limitations render this study not applicable to all persons of the general population.

## Conclusion

This study shows that green tea extract effectively increases leptin and reduces LDL in women after 6 weeks of treatment even though there were no significant changes in other biochemical markers related to overweight such as total cholesterol, triglyceride, and BMI.

## Additional files

Additional file 1: CONSORT Flow Diagram. (DOC 53 kb)

Additional file 2: This is the raw data that was gathered in the clinical trial and was used to establish the data base of this study. (DOC 219 kb)

## Abbreviations

BMI: Body mass index
COMT: Catechol-o-methyltransferase
CVD: Cardiovascular disease
EC: Epicatechin
ECG: Epicatechingallate
EGC: Epigallocatechin
EGCG: Epigallocatechin gallate
GTE: Green tea extract
LDL-C: Lipoprotein-cholesterol
WHO: World Health Organization
