# Supplementary material for: Effects of green tea extract on overweight and obese women with high levels of low density-lipoprotein-cholesterol (LDL-C): a randomised, double-blind, and cross-over placebo-controlled clinical trial
Source: BMC Complement Altern Med. 2018 Nov 6;18:294. doi: 10.1186/s12906-018-2355-x (PMC6218972; doi:10.1186/s12906-018-2355-x)
Supplement: Supplementary file 1 — CONSORT Flow Diagram. (DOC 53 kb) [file 12906_2018_2355_MOESM1_ESM.doc]

**
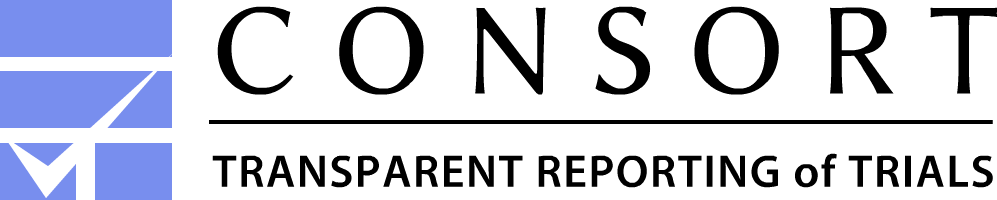
**

**CONSORT 2010 Flow Diagram**

**Analysis**

**Follow-Up 2**

Assessed for eligibility (n=236 )

Excluded (n=146 )

♦  Not meeting inclusion criteria (n=57)

♦  Declined to participate (n=89 )

♦  Other reasons (n= 0 )

Allocated to intervention (n=45)

♦ Received allocated intervention (n=45)

♦ Did not receive allocated intervention (n= 0 )

Allocated to intervention (n=45 )

♦ Received allocated intervention (n=45)

♦ Did not receive allocated intervention (n=0 )

**Allocation**

Randomized (n=90 )

**Enrollment**

**Follow-Up 1**

Analysed (n=36)
♦ Excluded from analysis (n=0)

Lost to follow-up (n=4)

- drop out

Discontinued intervention (n=0)

Lost to follow-up (n=3 )

- drop out

-loss of communication

Discontinued intervention (n=1)

- poor treatment response

Analysed (n=37)
♦ Excluded from analysis (n=0)

Lost to follow-up (n=3)

- drop out

Discontinued intervention (n=1)

- adverse effect

Lost to follow-up (n=4 )

- drop out

Discontinued intervention (n=1 )

- adverse effect
